# Biased M1 muscarinic receptor mutant mice show accelerated progression of prion neurodegenerative disease

**DOI:** 10.1073/pnas.2107389118

**Published:** 2021-12-10

**Authors:** Miriam Scarpa, Colin Molloy, Laura Jenkins, Bethany Strellis, Rebecca F. Budgett, Sarah Hesse, Louis Dwomoh, Sara Marsango, Gonzalo S. Tejeda, Mario Rossi, Zeshan Ahmed, Graeme Milligan, Brian D. Hudson, Andrew B. Tobin, Sophie J. Bradley

**Affiliations:** ^a^The Centre for Translational Pharmacology, Institute of Molecular, Cell and Systems Biology, College of Medical, Veterinary and Life Sciences, University of Glasgow, Glasgow G12 8QQ, UK;; ^b^Neuroscience Next Generation Therapeutics (NGTx), Eli Lilly and Company, Cambridge, MA 02142

**Keywords:** GPCR, M1 muscarinic acetylcholine receptor, phosphorylation, neurodegenerative disease

## Abstract

There are currently no treatments that can slow the progression of neurodegenerative diseases, such as Alzheimer’s disease (AD). There is, however, a growing body of evidence that activation of the M1 muscarinic acetylcholine receptor (M1-receptor) can not only restore memory loss in AD patients but in preclinical animal models can also slow neurodegenerative disease progression. The generation of an effective medicine targeting the M1-receptor has however been severely hampered by associated cholinergic adverse responses. By using genetically engineered mouse models that express a G protein–biased M1-receptor, we recently established that M1-receptor mediated adverse responses can be minimized by ensuring activating ligands maintain receptor phosphorylation/arrestin-dependent signaling. Here, we use these same genetic models in concert with murine prion disease, a terminal neurodegenerative disease showing key hallmarks of AD, to establish that phosphorylation/arrestin-dependent signaling delivers neuroprotection that both extends normal animal behavior and prolongs the life span of prion-diseased mice. Our data point to an important neuroprotective property inherent to the M1-receptor and indicate that next generation M1-receptor ligands designed to drive receptor phosphorylation/arrestin-dependent signaling would potentially show low adverse responses while delivering neuroprotection that will slow disease progression.

## Significance

The M1 muscarinic acetylcholine receptor (M1-receptor) plays a crucial role in learning and memory and is a validated drug target for the treatment of Alzheimer’s disease (AD). Furthermore, M1-receptor ligands have been demonstrated to display disease-modifying effects in preclinical models of neurodegenerative disease. By employing a genetic mouse model expressing a G protein–biased M1-receptor in combination with a mouse model of terminal neurodegenerative disease, we demonstrate here that the M1-receptor exerts an inherent neuroprotective activity that is dependent on its phosphorylation status. Thus, in AD drug development programs, M1-receptor ligands that maintain the receptor phosphorylation status will be more likely to lead to beneficial neuroprotective outcomes.

The number of people living with dementia, of which Alzheimer’s disease (AD) is the most common form, is estimated to be ∼50 million worldwide (1). This number is predicted to increase to around 130 million by 2050, in line with an aging population. Despite significant efforts to develop disease-modifying treatments for AD, there are currently no therapies that can slow or halt disease progression. Symptomatic treatment of memory loss in AD is currently available and delivered by cholinesterase inhibitors that aim to restore defective cholinergic transmission by elevating acetylcholine (ACh) levels in the brain. These drugs have limited clinical efficacy due mainly to dose-limiting side effects resulting from the nonselective whole-body up-regulation of cholinergic systems (2, 3). An alternative strategy is to directly activate ACh receptors of the muscarinic family, of which there are five subtypes (M1- to M5-receptors). Particular focus has been directed toward the M1 muscarinic acetylcholine receptor (M1-receptor) due to high levels of receptor expression in brain regions such as the hippocampus and cortex (4–6) and procognitive effects in preclinical animal models (7). Further, clinical trials using the orthosteric agonists, xanomeline and GSK-5, which primarily activate the M1-receptor have shown promising efficacy (8–10). Similarly, M1-receptor–selective positive allosteric modulators (PAMs) have been shown to improve cognition in preclinical animal models but together with the orthosteric ligands have ultimately failed in the clinic due largely to cholinergic adverse responses, some of which have been ascribed to on-target activity at the M1-receptor (11–13) as well as off-target M2- and M3-receptor activation (14, 15).

To overcome these barriers, we and others have set out to define the optimal pharmacological properties of orthosteric and allosteric M1-receptor ligands that will deliver clinical efficacy while minimizing cholinergic adverse responses (16). To this end, we have focused our attention on the possible advantages of biased ligands—an approach based on the observation that G protein–coupled receptors (GPCRs) operate by coupling to two fundamental signaling pathways: G protein–dependent signaling and receptor phosphorylation/arrestin-dependent pathways. The promise of ligand bias is that ligands could be designed to drive GPCR signaling pathways that lead to clinically beneficial outcomes, in preference to ones that result in adverse responses. We have investigated this possibility for the M1-receptor by the generation of a genetically engineered mouse strain that expresses a variant of the M1-receptor where all the intracellular phosphorylation sites have been removed (17). This variant (called M1-PD) is uncoupled from receptor phosphorylation/arrestin-dependent signaling but shows near normal coupling to Gq/11-dependent pathways. By using this receptor variant, we have established that cholinergic adverse responses to M1-receptor ligands are minimized if receptor phosphorylation/arrestin-dependent signaling is maintained (17). Furthermore, the M1-PD mice have established the importance of M1-receptor phosphorylation/arrestin-dependent signaling in the regulation of anxiety-like behaviors and learning and memory, suggesting that maintenance of receptor phosphorylation is important to deliver clinical efficacy as well as minimizing adverse responses.

Whereas early studies have provided a framework for the design of M1-receptor ligands for the symptomatic treatment for AD, it has been the emergence of evidence that the M1-receptor might also modify neurodegenerative disease progression that has generated significant attention (7, 18–20). Activation of muscarinic receptors with an orthosteric ligand can regulate the proteolytic processing of amyloid precursor protein, thereby reducing the appearance of amyloid-β (Aβ) plaques in a preclinical AD mouse model (19). Our own studies have established that M1-receptor–selective PAMs can slow the progression of mouse prion disease thereby maintaining normal animal behavior and extending the life span of terminally sick mice (18, 25).

Here, we have extended these studies by asking if the M1-receptor inherently has neuroprotective properties. By employing the M1-PD mouse strain (17) in combination with mouse prion disease, a progressive terminal neurodegenerative disease that shows many of the hallmarks of human AD (21), we report here that prion disease progresses more rapidly, and behavioral abnormalities appear at earlier times, in M1-PD mice compared to wild-type (WT) controls. The rapid disease onset in M1-PD mice is further evident in the elevation of neuroinflammatory pathways, including activation of astrocytes and microglia, and the up-regulation of markers of neurodegenerative disease. We conclude that the neuroprotective property of the M1-receptor might be harnessed by next generation M1-receptor drugs for the treatment of AD. M1-receptor–selective drugs could be designed to promote receptor signaling via phosphorylation/arrestin-dependent pathways, thereby not only delivering symptomatic relief in AD, by improving memory and reducing anxiety, but also delivering neuroprotection that will maintain normal behavior and extend life span.

## Results

### The M1-PD Receptor Is Coupled Normally to Gq/11 Signaling but Is Deficient in Arrestin Recruitment and Internalization.

**Fig. 1.**
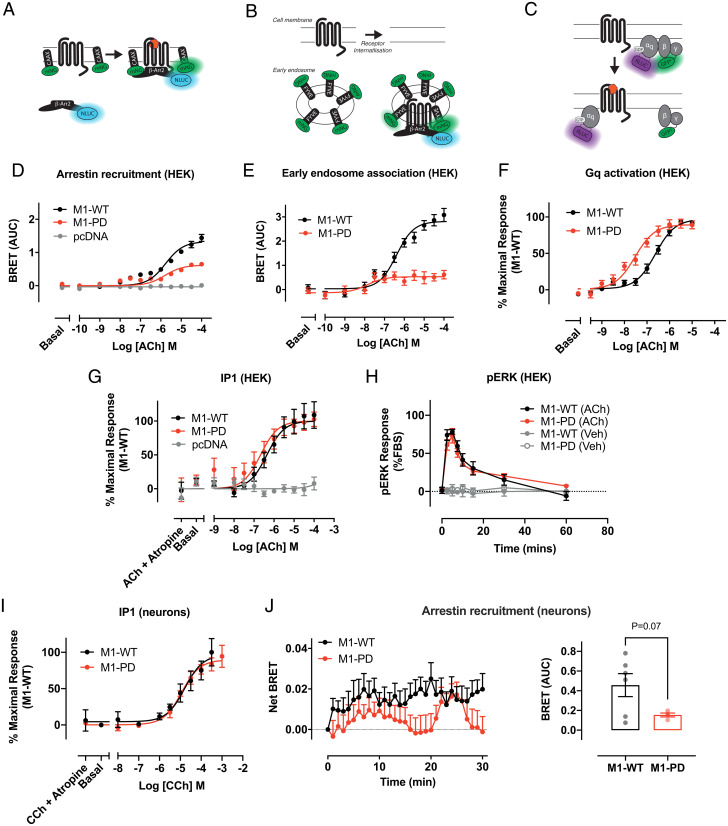
Arrestin recruitment and receptor internalization of the M1-receptor are dependent on receptor phosphorylation. (*A–C*) Schematic of the bystander BRET assays for arrestin recruitment to the M1-receptor (*A*), receptor translocation to early endosomes (*B*), and Gq activation to the M1-receptor (*C*). (*D*) ACh-stimulated translocation of β-arrestin-2 to the cell membrane in HEK293T cells transfected with pcDNA3, M1-WT, or M1-PD assessed by bystander BRET. Data are expressed as mean ± SEM of five independent experiments performed in triplicates. (*E*) Translocation of M1-WT and M1-PD to early endosomes in response to ACh treatment assessed through a bystander BRET assay. Data are expressed as mean ± SEM of five independent experiments performed in triplicates. (*F*) ACh-stimulated Gαq activation by the M1-WT or M1-PD receptor measured by a decrease in BRET. Data are expressed as means ± SEM of four independent experiments performed in quadruplicate. (*G*) IP1 accumulation after 60-min stimulation with ACh in HEK cells transiently transfected with the M1-WT and M1-PD constructs or the empty vector (pcDNA). Data are expressed as means ± SEM of four to seven independent experiments performed in duplicate or quadruplicate. (*H*) Time course of pERK signaling in HEK293T cells transfected with M1-WT or M1-PD and stimulated with ACh (100 μM) or vehicle (0.01% dimethyl sulfoxide). Data are expressed as means ± SEM of three independent experiments performed in triplicate (*n* = 3). (*I*) IP1 accumulation after 60-min stimulation with CCh in primary hippocampal-cortical neurons prepared from M1-WT or M1-PD mice. Data are expressed as means ± SEM of three independent experiments performed in duplicate. (*J*) Time course (*Left*) of ACh (100 μM)-stimulated translocation of β-arrestin-2 to the cell membrane in primary hippocampal-cortical neurons prepared from M1-WT or M1-PD mice. Mean area under the curve (AUC) is shown on the *Right*. Data are expressed as mean ± SEM of four to six independent experiments performed in triplicates.

Our previous studies established that removal of all mass spectrometry–identified phosphorylation sites of the M1-receptor (22) together with all other putative serine/threonine phosphorylation sites in the third intracellular loop and C-terminal tail, generated a mutant receptor (M1-PD) that is normally coupled to Gq/11 signaling, but deficient in arrestin recruitment (17). Here, we have extended our previous studies by employing BRET biosensor assays to measure β-arrestin recruitment and receptor internalization (Fig. 1 *A*, *B*, *D*, and *E*). In these experiments, HEK293 cells expressing the M1-PD receptor showed a reduction in ACh-mediated β-arrestin recruitment and receptor internalization compared to the WT receptor (Fig. 1 *D* and *E* and Table 1 and *SI Appendix*, Fig. S1 *A–F*). Using a BRET biosensor to measure Gq activation (Fig. 1*C*), we found that ACh stimulated Gq coupling to the M1-PD receptor with higher potency compared to the WT receptor (pEC50 = 6.4 ± 0.1 and 7.6 ± 0.1 for the WT and M1-PD, respectively), whereas the maximal response to ACh was equivalent (Fig. 1*F* and Table 1 and *SI Appendix*, Fig. S1 *G–I*). Coupling to signaling pathways downstream of Gq activation, inositol phosphate accumulation (Fig. 1*G* and Table 1) and ERK1/2 phosphorylation (pERK1/2) (Fig. 1*H*) was equivalent between M1-PD and WT receptors. Furthermore, our data demonstrate that the pERK1/2 response is mediated entirely by Gq protein-dependent signaling, since preincubation with a Gq inhibitor completely abolished the response to ACh at both the M1-WT and M1-PD receptor (*SI Appendix*, Fig. S2). By fitting the ACh concentration response curves for Gq activation or arrestin recruitment to the operational model of agonism, we derived a transduction coefficient (τ) for each of these responses at the M1-PD receptor. We compared these values with the τ calculated for each of these pathways at the M1-WT and calculated a bias factor [ΔΔlog10(τ/KA)]. These analyses showed that M1-PD receptors show preferential signaling bias (bias factor = 20.66) toward Gq activation versus arrestin recruitment (Table 2).

**Table 1.** M1-PD shows equivalent G protein–mediated responses compared to M1-WT but impaired arrestin recruitment and receptor internalization

		pEC_50_	E_max_	*n*
Arrestin recruitment (HEK)	M1-WT	5.7 ± 0.1	104.0 ± 1.0	5
	M1-PD	5.4 ± 0.1	40.7 ± 2.4***	5
Internalization (HEK)	M1-WT	6.4 ± 0.1	92.5 ± 2.1	5
	M1-PD	5.8 ± 1.9	22.5 ± 5.4***	5
Gq activation (HEK)	M1-WT	6.4 ± 0.1	100.0 ± 0.0	5
	M1-PD	7.6 ± 0.1***	108.0 ± 7.5	5
IP1 (HEK)	M1-WT	6.4 ± 0.1	107.5 ± 7.3	7
	M1-PD	6.7 ± 0.2	98.6 ± 7.3	5
IP1 (neurons)	M1-WT	4.8 ± 0.2	96.3 ± 9.5	3
	M1-PD	4.9 ± 0.1	89.6 ± 4.3	3

Potency and maximum effect of agonist stimulated β-arrestin-2 recruitment, receptor translocation to early endosomes, Gq activation, and IP1 accumulation at M1-WT or M1-PD receptors. Agonists used for HEK cells and neurons were ACh or CCh, respectively. Data are expressed as the means ± SEM of three to seven independent experiments. Data were analyzed using an unpaired *t* test, where ***P < 0.001 compared to WT.

**Table 2.** Bias calculations for Gq activation or arrestin recruitment at the M1-PD receptor

	Gq activation		Arrestin recruitment		Log bias factor Gq − arrestin
	Log_10_(τ/K_A_)	ΔLog_10_(τ/K_A_)	Log_10_(τ/K_A_)	ΔLog_10_(τ/K_A_)	ΔΔLog_10_(τ/K_A_)
M1-WT	6.4 ± 0.08	0.00 ± 0.11	6.12 ± 0.29	0.00 ± 0.40	0.00 ± 0.42
M1-PD	7.36 ± 0.10	0.95 ± 0.12	5.76 ± 0.13	−0.36 ± 0.32	1.32 ± 0.34

M1-PD receptors expressed in HEK cells show preferential signaling bias [ΔΔlog10(τ/KA)] toward Gq activation versus arrestin recruitment using BRET biosensors. Data are presented as means ± SEM, with M1-WT receptors used as the reference. Pairwise comparisons between Gq activation versus arrestin recruitment at the M1-WT and M1-PD were performed using unpaired Student’s *t* test. No significant difference was observed between logτ/ΚΑ values obtained in Gq versus arrestin assays for M1-WT, but a significant difference between the assay logτ/ΚΑ values (*P* < 0.0001) was observed for M1-PD.

We have previously described the generation of genetically engineered mice where the coding sequence for the M1-PD variant was knocked-in into the natural gene locus of the M1-receptor (chrm1) (17). In addition, to aid identification of receptor expression a hemagglutinin (HA) epitope tag was fused to the C terminus of the M1-PD receptor. Control mice were also generated where the coding sequence for the mouse WT M1-receptor fused at the C terminus with an HA-tag was similarly knocked into the M1-receptor locus (these mice are termed M1-WT mice). Consistent with previous studies (17), we show here that M1-PD transcription in the cortex and hippocampus of M1-PD mice is comparable to that of the M1-receptor in M1-WT mice (*SI Appendix*, Fig. S3*A*). Furthermore, quantification of receptor expression using Western blotting to detect the HA-tag revealed no significant (*P* > 0.05) difference in receptor expression when comparing M1-WT and M1-PD mice (*SI Appendix*, Fig. S3 *B* and *C*). Consistent with our findings in recombinant systems, we also show here that in primary hippocampal-cortical neurons prepared from M1-WT or M1-PD mice, agonist-stimulated inositol phosphate accumulation is equivalent (Fig. 1*I* and Table 1). Finally, by transfection of neurons with mNG-CAAX and β-Arrestin-2 fused to nanoluciferase, we were able to measure arrestin recruitment to endogenously expressed M1-receptors. These studies demonstrated a reduction in the maximal ACh-stimulated arrestin recruitment to the M1-PD compared to the M1-WT (Fig. 1*J*). As a positive control, neurons were also transfected with mRNA coding for the human free fatty acid 4 (FFA4) receptor, a GPCR known to interact strongly with β-arrestin-2 (23). Stimulation with TUG-891, an FFA4 receptor agonist, led to comparable arrestin recruitment to FFA4 receptors expressed in neurons prepared from M1-WT and M1-PD transgenic mice (*SI Appendix*, Fig. S4 *A* and *B*).

### Prion-Infected M1-PD Mice Show Key Hallmarks of Disease Earlier than M1-WT Mice.

**Fig. 2.**
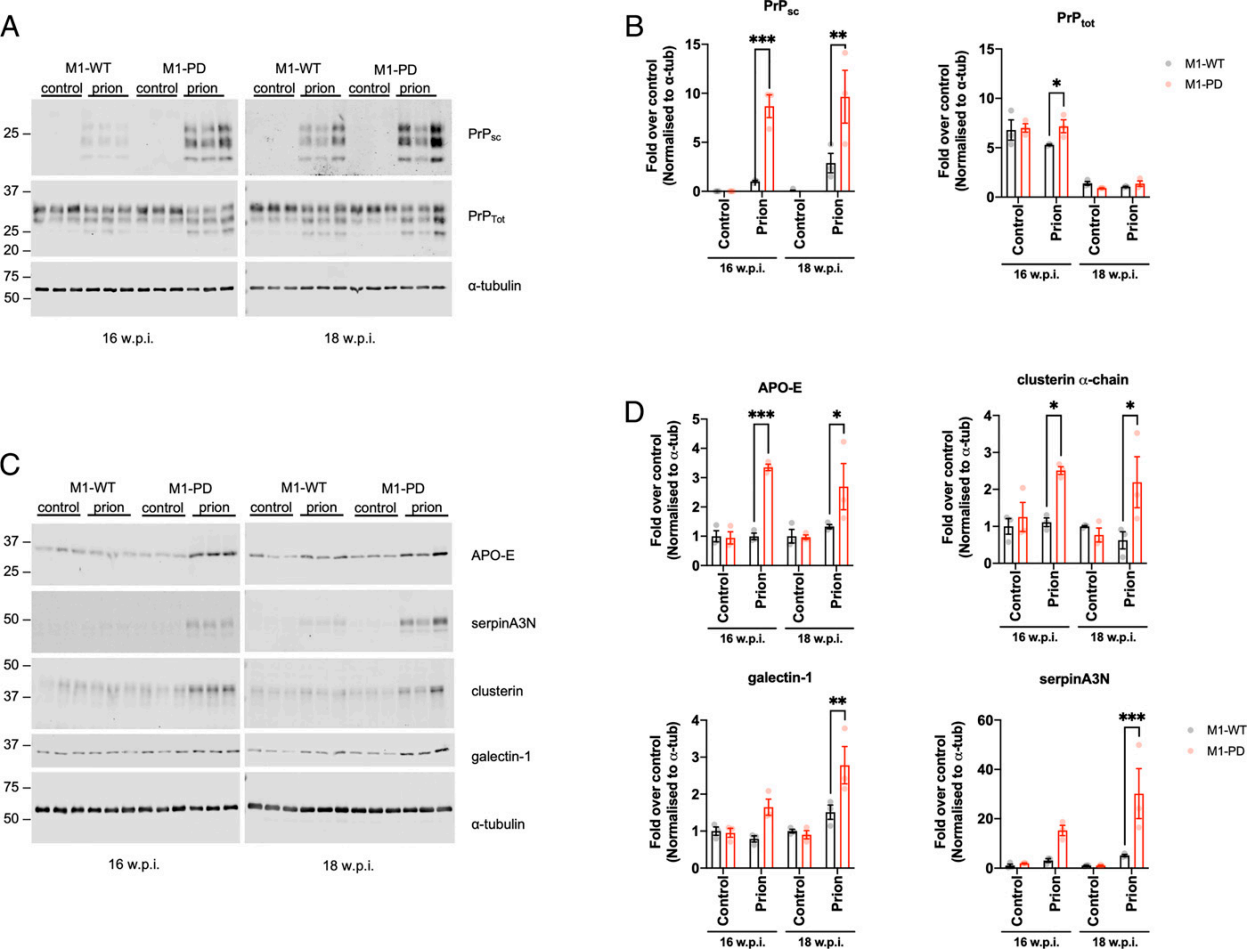
Prion-infected M1-PD mice show accelerated appearance of disease markers in the hippocampus compared to M1-WT mice. Lysates were prepared from the hippocampus of control or prion-infected M1-WT and M1-PD mice at 16 and 18 w.p.i., and Western blot analysis was used to analyze the expression of a panel of pathological markers. (*A*) Lysates were incubated in the presence or absence of proteinase K prior to Western blot to detect nondigested scrapie prion protein (PrP_sc_) and total prion protein (PrP_tot_), respectively. Band analysis for PrP_sc_ and PrP_tot_ expression in (*B*) is shown as means ± SEM of a ratio of α-tubulin expression. (*C*) APO-E, serpinA3N, clusterin, and galectin-1 were detected in the hippocampus and band analysis is shown in *D* as means ± SEM of a ratio of α-tubulin expression relative to control-infected M1-WT (*n* = 3 mice). All data were analyzed using two-way ANOVA with Sidak multiple comparisons where *P < 0.05, **P < 0.01, ***P < 0.001 (M1-WT versus M1-PD).

Our previous work demonstrated that M1-receptor PAMs can offer both symptomatic and disease-modifying properties in a mouse model of terminal neurodegenerative disease (18). Mice inoculated with Rocky Mountain Laboratory (RML) prion-infected brain homogenate develop terminal neurodegenerative disease showing progressive neuronal loss, significant neuroinflammation and behavioral deficits. Here, we aimed to define the role of M1 receptor phosphorylation/arrestin-dependent signaling pathways in neurodegenerative disease progression. M1-WT or M1-PD mice were inoculated with control (normal, healthy brain homogenate) or prion-infected brain homogenates, and receptor expression levels were found to be unchanged in control and prion-diseased mice at 16 weeks postinoculation (w.p.i.) (*SI Appendix*, Fig. S5 *A–C*). Inoculation of mice with prion-infected brain homogenate induces accumulation of misfolded, insoluble prion protein that is resistant to digestion with proteinase K (PrPsc) (18, 21, 24). M1-PD mice inoculated with prion-infected brain homogenate show earlier increases in PrPsc accumulation in the hippocampus and cortex compared to M1-WT mice with prion disease (Fig. 2 *A* and *B* and *SI Appendix*, Fig. S6 *A* and *B*). Since the time course of prion disease progression is dictated by the expression level of cellular prion protein (PrPc), whereby increased PrPc expression accelerates disease progression (21), we assessed PrPc expression in M1-PD mice. Importantly, we found that PrPc transcript levels were equivalent in M1-WT, M1-PD, and M1-KO mice (*SI Appendix*, Fig. S7). These data show that prion disease had progressed faster in mice expressing a variant of the M1-receptor with reduced coupling to phosphorylation/arrestin signaling, suggesting that M1-receptor signaling through this pathway mediates a previously unappreciated neuroprotective effect.

We wanted to further test the possibility that uncoupling the M1-receptor from phosphorylation/arrestin signaling removed a neuroprotective component of receptor activity by monitoring biomarkers of disease severity. Recently, we have mapped the pathological changes in prion disease by conducting global transcriptomic and proteomic analyses of the hippocampus of prion-diseased mice. We have found several protein markers previously associated with human AD that are significantly up-regulated in prion disease. These included markers of neuroinflammation, glial fibrillary acidic protein (GFAP), clusterin, vimentin, and galectin-1 and markers of adaptive responses to neurodegeneration including apolipoprotein-E (APO-E) and the protease inhibitor serpinA3N (25). In this study, we suggested that the up-regulation of these proteins represent biomarkers of prion disease and indicators of disease severity (25). Here, we used the expression of these proteins to monitor disease progression in prion-infected M1-WT and M1-PD mice. Whereas M1-WT mice showed little change in the expression of APO-E, serpinA3N, clusterin α-chain, and galectin-1 at 16 and 18 w.p.i., M1-PD mice showed a significant increase in all four of these prion disease biomarkers in the hippocampus and/or cortex (Fig. 2 *C* and *D* and *SI Appendix*, Fig. S6 *C* and *D*). These results provide further evidence that the M1-PD variant of the M1-receptor allows accelerated prion-induced pathological changes. Importantly, we show that acceleration of disease in M1-PD mice is not due to the detrimental activity of sustained ERK1/2 signaling (26) since ERK1/2 expression and activation was comparable in control and prion-diseased M1-WT and M1-PD mice (*SI Appendix*, Fig. S8). However, we did detect a nonsignificant trend for reduced phosphorylation of ERK1/2 in the hippocampus of prion-infected M1-PD versus M1-WT mice, which may warrant further investigations.

**Fig. 3.**
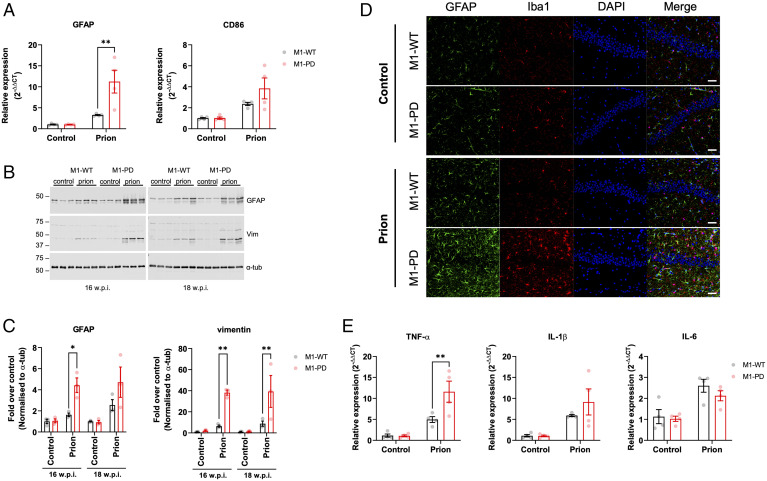
Neuroinflammation is exacerbated in the hippocampus of prion-infected M1-PD mice compared to M1-WT controls. (*A*) mRNA levels of GFAP and CD86, markers of astrocytes and microglia, respectively, were quantified using quantitative RT-PCR of hippocampus from control or prion-diseased M1-WT or M1-PD mice at 16 w.p.i. Data are expressed as means ± SEM of a ratio of α-tubulin RNA expression relative to M1-WT (*n* = 4 mice). **P < 0.01, two-way ANOVA with Sidak multiple comparisons (M1-WT versus M1-PD). (*B* and *C*) Astrogliosis in the hippocampus was assessed using Western blot analysis of lysates prepared from control or prion-infected mice at 16 and 18 w.p.i. Lysates were probed for astrocytic markers GFAP and vimentin (vim), and α-tubulin (α-tub) antibody was used as a loading control. (C) Band analysis for each blot was performed, and data are shown as means ± SEM of a ratio of α-tubulin relative to control M1-WT (*n* = 3 mice). *P < 0.05, **P < 0.01, two-way ANOVA Sidak multiple comparisons (M1-WT versus M1-PD). (*D*) Immunohistochemical staining for GFAP and Iba-1 in the hippocampus of control and prion-infected M1-WT and M1-PD mice at 16 w.p.i. The nuclei were stained blue with DAPI. (Scale bar, 100 μm.) (*E*) Quantitative RT-PCR showing the expression of proinflammatory (TNF-α, IL-1β, IL-6) cytokines in the hippocampus of control and prion-infected M1-WT and M1-PD mice at 16 w.p.i. Data are expressed as a ratio of α-tubulin RNA expression relative to control M1-WT (*n* = 4 mice). Data were analyzed using two-way ANOVA with Sidak’s multiple comparisons, where **P < 0.01 (M1-WT versus M1-PD).

### Prion-Infected M1-PD Mice Show Increased Neuroinflammation Compared to M1-WT Mice.

Murine prion disease, similar to many human neurodegenerative diseases (27), is associated with profound neuroinflammation characterized by activation of both astrocytes and microglia (28). Here, we further investigated the severity of prion disease in M1-WT and M1-PD mice by assessing the status of neuroinflammation. In prion-diseased M1-PD mice, transcripts for GFAP and CD86, markers for astrocytes and microglia, respectively, were significantly elevated compared to M1-WT mice (Fig. 3*A* and *SI Appendix*, Fig. S9*A*). Furthermore, Western blotting for GFAP and vimentin revealed an up-regulation of astroglia in the hippocampus and cortex of prion-infected M1-PD mice (Fig. 3 *B* and *C* and *SI Appendix*, Fig. S9 *B* and *C*). Importantly, the levels of both GFAP and vimentin were significantly higher in the hippocampus of prion-inoculated M1-PD mice compared to M1-WT mice (Fig. 3 *A*–*C*). Protein levels of GFAP and vimentin trended higher in the cortex of M1-PD mice infected with prion compared to the respective WT animals at 18 w.p.i. (*SI Appendix*, Fig. S9 *B* and *C*).

We further assessed the status of neuroinflammation by immunohistochemical staining of sections of the hippocampus and cortex. Staining for GFAP (astrocytes) and Iba-1 (microglia) demonstrated a profound increase in neuroinflammatory markers in the hippocampus and cortex of M1-PD, compared to M1-WT (Fig. 3*D* and *SI Appendix*, Fig. S9*D*). Transcription of the proinflammatory cytokines TNF-α, IL-1β, and IL-6 was elevated in the hippocampus and cortex of prion-infected M1-WT mice compared to M1-WT mice inoculated with normal brain homogenate (Fig. 3*E* and *SI Appendix*, Fig. S9*E*). Furthermore, we detected a significant increase in transcription of TNF-α in the hippocampus and cortex of M1-PD prion mice compared to M1-WT, whereas IL-1β levels were elevated in the M1-PD versus M1-WT in the cortex only (Fig. 3*E* and *SI Appendix*, Fig. S9*E*).

There were no differences in the expression of transcripts for antiinflammatory cytokines, IL-4, IL-10, IL-11, and IL-13, in the cortex or hippocampus of prion-infected M1-WT or M1-PD mice (*SI Appendix*, Fig. S10). Importantly, expression of transcripts for GFAP, CD86, and the battery of pro- and antiinflammatory cytokines tested previously were equivalent in noninfected M1-WT and M1-PD mice (*SI Appendix*, Fig. S11). Taken together, these data indicate that prion-infected M1-PD mice show increased neuroinflammation compared to M1-WT mice.

### M1-PD Leads to Earlier Disease Onset and Shorter Survival Time.

**Fig. 4.**
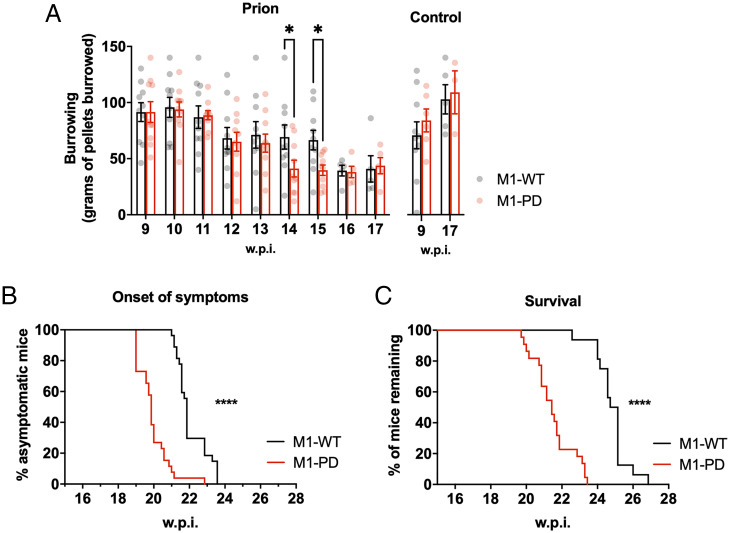
Removal of M1-receptor phosphorylation sites accelerates prion disease and decreases survival time. (*A*) Burrowing responses (food pellets [grams] displaced from the tube) of control or prion-infected M1-WT and M1-PD mice were assessed from 9 w.p.i. (*n* = 3 to 10 mice; *P < 0.05; two-way ANOVA or mixed-effects model with uncorrected Fisher’s least significant difference test). Onset of at least two early indicators of prion disease (*n* = 26 to 27) (*B*) and Kaplan–Meier survival plot (*n* = 16 to 22) (*C*) for prion-infected M1-WT and M1-PD. Curves were analyzed with a Gehan–Breslow–Wilcoxon test, where ****P < 0.0001.

Prion-diseased mice exhibit behavioral deficiencies in burrowing activity, as reported previously (24), indicating a decline in hippocampal function. We assessed burrowing behavior in prion-infected M1-WT and M1-PD mice and found that the M1-PD mice exhibited accelerated decline in burrowing ability. Specifically, the burrowing behavior was significantly reduced at 14 (*P* = 0.048) and 15 w.p.i. (*P* = 0.017) in prion-diseased M1-PD mice when compared to the M1-WT (Fig. 4*A*). From 16 w.p.i. onwards, there were no significant differences in burrowing behavior of prion-infected M1-WT and M1-PD mice. Importantly, the burrowing responses of M1-WT and M1-PD mice inoculated with control brain homogenate were equivalent at 9 and 17 w.p.i.

Finally, we assessed the impact of M1-receptor phosphorylation on survival of prion-diseased mice. Murine prion disease is a terminal neurodegenerative disease where clinical signs of disease—subdued behavior, intermittent generalized tremor, erect penis, rigid tail, unsustained hunched posture, and mild loss of coordination—are evident from ∼21 w.p.i. in M1-WT mice (*SI Appendix*, Fig. S12). The appearance of at least two of the early indicator signs of prion disease occurred significantly earlier (*P* < 0.0001) in M1-PD mice compared to M1-WT mice (Fig. 4*B*). The median time for the onset of symptoms was 22 w.p.i. for M1-WT mice and 20 w.p.i. for M1-PD mice. Mice expressing the phosphorylation-deficient M1-receptor mutant showed a significant (*P* < 0.0001) acceleration of confirmatory scrapie diagnosis, with median time for terminal illness of 25 w.p.i. for M1-WT mice and 21 w.p.i. for M1-PD mice (Fig. 4*C*). These data demonstrate that in addition to showing accelerated onset of behavioral abnormalities (burrowing), the M1-PD mice also showed an acceleration of the onset of clinical symptoms of prion disease.

## Discussion

By genetically engineering mice to express a phosphorylation-deficient variant of the M1-receptor, we demonstrate here that physiological M1-receptor coupling to phosphorylation/arrestin-dependent pathways provides protection from prion-induced neurodegeneration. This was evident in the rapid onset of prion disease in mice expressing a variant of the M1-receptor (M1-PD) that has all potential phosphorylation sites removed (17). Previous studies from our laboratory have focused on the outcome of pharmacologically targeting the M1-receptor in prion disease. These earlier studies demonstrated that exogenous ligands that activate the M1-receptor can restore learning and memory deficits in prion disease and slow disease progression (18). Here, we extend these studies by revealing an inherent endogenously regulated M1-receptor neuroprotective activity that results in suppression of markers of neurodegenerative disease and a reduction in neuroinflammation. Furthermore, our data show that this neuroprotective mechanism depends on the receptor phosphorylation status.

Our study has important implications for drug design. Like many members of the GPCR superfamily, muscarinic receptors mediate signal transduction in a bimodal fashion that involves both canonical G protein signaling and receptor phosphorylation/arrestin-dependent signaling. Whereas the molecular details of this phenomenon have been extensively studied in in vitro transfected cell systems, understanding the physiological importance of bimodal signaling and, further, how this might have a pathophysiological impact has been extremely challenging. We have approached this problem by genetically engineering mice to express variants of receptors where intracellular phosphorylation sites have been removed, thereby reducing coupling to arrestin but maintaining coupling to heterotrimeric G proteins. This approach has been successfully employed, for example, in mapping physiological processes downstream of the M3-receptor (29). Applying this same approach to the M1-receptor, a receptor widely considered as a validated target for improving memory loss and promising disease-modification in AD (7), our previous studies have established that activating ligands that maintain receptor phosphorylation/arrestin-dependent signaling may deliver clinically relevant efficacy, such as mediating procognitive effects,, while minimizing cholinergic adverse responses (17). Our study here extends these observations and suggests that M1-receptor ligands designed to promote receptor phosphorylation will have the additional benefit of driving neuroprotective receptor activity.

Our study also has important pathophysiological implications. AD is characterized by a loss of cholinergic neurons originating from the basal forebrain innervating the neocortex, amygdala, hippocampus, and entorhinal cortex (30–32). The loss of cholinergic innervation is thought to be responsible for cognitive deficits in AD (31, 33), a hypothesis that underpins the use of cholinesterase inhibitors as the primary clinical strategy to improve memory loss in AD. The discovery here that M1-receptor activity has an inherent neuroprotective effect suggests that progressive loss of cholinergic neurons in AD will not only result in the loss of transmission in key memory centers but also a concurrent diminution of the M1-receptor neuroprotective effect. In this way, the loss of cholinergic neurons in AD with the associated reduction in ACh-driven activation of postsynaptic M1-receptors might contribute to disease progression.

Global proteomic and transcriptomic studies have established that murine prion disease shows many of the key hallmarks of AD including neuroinflammation, mitochondrial dysfunction, and oxidative stress (25). Furthermore, adaptive processes associated with the clearance of misfolded proteins commonly seen to be up-regulated in AD are also seen to be up-regulated in murine prion disease (25). These studies support the notion that neurodegenerative diseases propagated by the spread of “prion-like” misfolded proteins share common disease features (34). It is therefore significant that disease markers common to AD and prion disease such as APO-E and serpinA3N as well as markers of neuroinflammation are seen to be elevated in M1-PD prion-infected mice. This indicates that the neuroprotection resulting from endogenous M1-receptor phosphorylation/arrestin-dependent signaling could be relevant to the protection against other neurodegenerative diseases that result from the accumulation of “prion-like” misfolded proteins.

One possibility is that lack of receptor phosphorylation could reduce the desensitization of GPCR signaling, resulting in excessive activation of signaling pathways (35, 36). For example, excessive and sustained activation of ERK1/2 signaling has been associated with neuronal cell death in neurodegenerative diseases such as AD, attributed to oxidative stress (37, 38), hyperphosphorylation of tau (39–41), and Aβ toxicity (42, 43). We have established here, however, that uncoupling the M1-receptor from phosphorylation/arrestin-dependent signaling has no impact on ERK1/2 signaling both in recombinant signaling assays and in samples prepared from the brains of control or prion-diseased M1-WT and M1-PD mice. Our data therefore indicate that the accelerated neurodegeneration observed in prion-diseased M1-PD mice is not due to an overall excessive activation of the ERK1/2 pathway but instead caused by the removal of an alternative neuroprotective mechanism that is dependent on M1-receptor phosphorylation.

Although receptor phosphorylation is important to stabilize the interaction with arrestins (44), β-arrestins can interact with GPCRs in a phosphorylation-dependent and independent manner (44–47) whereby receptor-arrestin complexes are found as partially engaged (only bound through the receptor phosphorylated C-tail) and/or fully engaged complexes (bound through receptor core and tail) (48, 49). In particular, the discovery of the partially engaged receptor-arrestin complex suggests that it might be possible for a GPCR to be able to engage with both arrestins and G proteins simultaneously. It was demonstrated in vitro that GPCRs can form megaplexes with Gαs subunits and β-arrestins when internalized (50). Thus, removal of receptor phosphorylation can significantly impact multiple downstream signaling pathways and mechanisms by altering receptor interactions with signaling partners and consequential active conformations of arrestins. Therefore, removal of the M1-receptor phosphorylation sites could not only affect receptor desensitization, arrestin recruitment, and trafficking as shown here but could also influence other possible M1-receptor mediated mechanisms by shifting to specific arrestin active conformations and altering signaling transduction by G protein–arrestin megaplexes (51–53).

In conclusion, we establish that the M1-receptor has inherent neuroprotective activity that results in the suppression of neuroinflammation and diminishes markers of neurodegenerative disease and is driven by a mechanism that requires receptor phosphorylation. Our data suggest that M1-receptor ligands that promote receptor phosphorylation signaling will not only act to improve memory deficits in neurodegenerative diseases such as AD but will deliver neuroprotection that will maintain normal behavior and extend life span.

## Materials and Methods

### Mouse Maintenance and Diet.

Generation of the M1-WT, M1-PD, and M1-KO strains used was described previously (17). All mice were bred and maintained as homozygous colonies on a C57BL/6J background, with backcrossing onto C57BL/6J WT mice performed after 10 generations. M1-WT and M1-PD knock-in lines express WT and phosphorylation-deficient mutant forms of the M1 receptor, respectively, but both have an HA-tag appended to the C terminus. Experiments in noninfected mice were conducted on male and female mice at 8 to 12 wk old. Mice were fed ad libitum with a standard mouse chow. Animals were cared for in accordance with national guidelines on animal experimentation, and all experiments were conducted under a UK Home Office project license under the Animals (Scientific Procedures) Act (1986).

### Primary Neuronal Culture.

Tissue culture plates were coated using 4 µg/mL poly-D-lysine and 6 µg/mL Laminin Mouse Protein in diethyl pyrocarbonate (DEPC)–treated H_2_O and incubated overnight at 37 °C. Plates were then washed three times using DEPC-treated H_2_O and dried for 2 h at room temperature.

The hippocampal and cortical areas of the brain were isolated from E16 mouse embryos. The tissues were chopped into smaller pieces, washed three times in Hanks’ balanced salt solution, transferred to a 15 mL tube containing 4 mL of TrypLE Select 10X, and incubated at 37 °C for 10 min. TrypLE Select 10X was then inactivated by adding 8 mL of neurobasal complete media (Neurobasal Plus medium supplemented with 20 mL/L B-27 Plus, 0.292 g/L L-glutamine, 100 U/mL penicillin, 0.1 mg/L streptomycin) to the tubes followed by centrifugation at 200 × *g* for 5 min. The pellet was resuspended in neurobasal complete media to a final density of 5 × 10^5^ cells/mL. Cells were then seeded onto precoated plates and maintained at 37 °C in a 5% CO_2_ humidified atmosphere.

### Prion Infection of Mice.

Transgenic knock-in mice expressing HA-tagged M1-WT and M1-PD receptors were inoculated by intracerebral injection (under 3% isoflurane anesthesia) into the right parietal lobe with 1% brain homogenate infected with RML prion aged 3 to 4 wk as described previously (18). Control mice received 1% normal brain homogenate.

### Burrowing.

Assessment of burrowing on control and prion-infected M1-WT or M1-PD mice was conducted from 9 w.p.i. The burrowing test involved mice being placed into individual cages (22 × 36 cm) with a plastic cylinder filled with 140 g of food pellets. Food remaining in the cylinders after 2 h was weighed and the amount displaced (“burrowed”) was calculated. Prior to the burrowing test, mice were placed in the burrowing cage for a 2-h period. The test was then repeated on a weekly basis.

### Symptoms Scoring and Survival Analysis.

Prion-infected mice were scored according to the appearance of recognized early indicator and confirmatory signs of prion disease. Early indicator signs included piloerection, sustained erect ears, erect penis, clasping of hind legs when lifted by tail, rigid tail, unsustained hunched posture, mild loss of coordination, or being subdued. Confirmatory signs of prion disease included ataxia, impairment of a righting reflex, dragging of limbs, sustained hunched posture, and/or significant abnormal breathing. Survival times were calculated based on the presence of two early indicator signs plus one confirmatory sign or two confirmatory signs. At this time, mice would be humanely killed.

## Supplementary Material

Supplementary File

## Data Availability

Raw data have been deposited in University of Glasgow Enlighten repository (https://dx.doi.org/10.5525/gla.researchdata.1202). All other study data are included in the article and/or *SI Appendix*.
