# Supplementary material for: Biased M1 muscarinic receptor mutant mice show accelerated progression of prion neurodegenerative disease
Source: Proc Natl Acad Sci U S A. 2021 Dec 10;118(50):e2107389118. doi: 10.1073/pnas.2107389118 (PMC8685681; doi:10.1073/pnas.2107389118)

Supplementary Information for:

Biased M1 muscarinic receptor mutant mice show accelerated progression of prion neurodegenerative disease

Miriam Scarpa[1], Colin Molloy[1], Laura Jenkins[1], Bethany Strellis[1], Rebecca Budgett[1], Sarah Hesse[1], Louis Dwomoh[1], Sara Marsango[1], Gonzalo Tejeda[1], Mario Rossi[1], Zeshan Ahmed[2], Graeme Milligan[1], Brian D. Hudson[1], Andrew B. Tobin[1] and Sophie J. Bradley[1,3].

[1] The Centre for Translational Pharmacology, Institute of Molecular, Cell and Systems Biology, College of Medical, Veterinary and Life Sciences, University of Glasgow, Glasgow, G12 8QQ, UK.

[2] Eli Lilly & Co, Neuroscience Discovery, Erl Wood Manor, Windlesham, Surrey, GU20 6PH, UK.

[3] Sosei Heptares, Steinmetz Building, Granta Park, Great Abingdon, Cambridge, UK.

Corresponding authors:

Sophie J. Bradley; sophie.bradley@glasgow.ac.uk

Andrew B. Tobin; andrew.tobin@glasgow.ac.uk

This PDF file includes:

Supplementary Methods

SI References

Figures S1-S12

Legends for Figures S1-S12

Supplementary Methods

Human Embryonic Kidney (HEK) cell culture

HEK293T cells were maintained in Delbecco's Modifeid Eagle Medium (DMEM) containing 10% FBS and maintained at 37°C and 5% CO_2. HEK293T cells were transfected with polyethylenimine (PEI), using a 1:6 weight to weight ratio of DNA to PEI. 24 h post transfection cells were detached and seeded at either 20,000 cells/well into poly-D-lysine coated white 96-well plates for BRET assays, or 40,000 cells/well into poly-D-lysine coated clear 96-well plates for IP1 assay. Cells were then returned to the incubator and cultured for a further 24 h for BRET based assays or 48 h for IP1 accumulation assays.

IP1 Accumulation assay

HEK293T cells transiently transfected with vectors containing the M1-WT or M1-PD, or with the empty vector (pcDNA) were plated on 96-well plates at cell density of 40,000 cells/well for IP1 accumulation assays. Cells were washed and incubated in 1X stimulation buffer (Hank's Balanced Salt Solution (HBSS) w/o phenol red containing 20 mM HEPES, 1.2 mM $CaCl_2$; 30 mM LiCl; pH7.4) for one hour at 37°C prior drug treatments. 10X concentrated test compounds were added (5 µl/well) to the 96-well plates and incubated at 37°C for one hour. Following the treatment, the stimulation buffer was removed and lysis buffer (IP-One assay kit, CisBio) was added (40 µl/well). Following 10 minute-incubation on a shaker at 600 rpm, cell suspensions (7µl/well) were added to 384-well white proxiplates (PerkinElmer). The IP1-d2 conjugate and the anti-IP1 cryptate Tb conjugate (IP-One Tb™ assay

kit, CisBio) were diluted together 1:40 in lysis buffer and 3 µl of the antibody mix were added to each well. The plate was incubated at 37°C for 1-24h and FRET between d2-conjugated IP1 (emission at 665 nm) and Lumi4™-Tb cryptate conjugated anti-IP1 antibody (emission at 620 nm) was detected using a CLARIOstar plate reader (BMG Labtech). Results were calculated from the 665/620 nm ratio and normalised to the maximum response stimulated by ACh.

Primary neuronal cells were seeded at a density of 5×10^4 cells per well onto pre-coated 96 well plates and maintained at 37 °C in a 5% CO_2 humidified atmosphere. On DIV7 cells were washed and incubated in stimulation buffer, and the assay was conducted as detailed above. Inhibition of agonist stimulation was measured by pre-incubating cells with 10 µM atropine for 30 min at 37 °C prior to addition of carbachol for 1 hour.

ERK 1/2 phosphorylation assay

HEK293T cells transiently transfected with vectors containing the M1-WT or M1-PD were plated on 96-well plates at cell density of 50,000 cells/well for ERK phosphorylation assay using the CisBio Phospho-ERK (Thr202/Tyr404) cellular kit (PerkinElmer). After incubation overnight at 37 °C, the culture medium was replaced with serum-free DMEM, and the cells were incubated for a further 4-5 hours prior to the assay. To inhibit Gq signalling, cells were pre-incubated with 1 µM FR900359 (1) for 1 hour. For the assay, cells were stimulated with FBS, vehicle (0.1% DMSO), or 1 µM acetylcholine for 8 different time points (0, 2.5, 5, 7.5, 10,

15, 30 and 60 minutes) at 37 °C. The reactions were terminated by the removal of the compounds and the addition of lysis buffer supplemented with blocking buffer. The lysates were shaken (600 rpm) for 10 minutes at room temperature before being transferred to a 384-well white OptiPlate (PerkinElmer) where they were incubated with a premixed antibody solution (anti-phospho-ERK1/2 Cryptate and anti-phospho-ERK1/2 d2) for 1-24 hours at room temperature with agitation at 600 rpm. Fluorescence emission (620 nm and 665 nm) was determined using a CLARIOstar plate reader (BMG Labtech) and results normalised to the response stimulated by FBS.

BRET arrestin assay

HEK cells

To assess arrestin recruitment to M1-WT and M1-PD, a bystander BRET assay was employed (2). HEK293T cells were co-transfected with plasmids encoding: 1) human β-Arrestin-2 fused to Nanoluciferase (Nluc) at its N-terminus; 2) the mNeonGreen (mNG) fluorescent protein fused with the prenylation CAAX sequence of KRas at its C-terminus; and 3) Either an empty pcDNA3 plasmid, a plasmid encoding wild-type mouse M1, or a plasmid encoding mouse M1-PD. For these transfections a DNA weight ratio of 1:25:5 was used for the Nluc-β-Arrestin-2 : mNG-CAAX : pcDNA3/M1-WT/M1-PD plasmids. After transfection, cells were cultured 24h, transferred to white 96-well plates and cultured a further 24h. For the assay, transfected cells in white 96-well plates were first washed twice with HBSS supplemented with 20mM HEPES (HBSS-H) and incubated for 30 min at 37°C.

Nluc substrate, Coelenterazine 400a, was then added to a final concentration of 5 μM and incubated for 10 min. Dual 535 and 475 nm luminescent emission measurements were then taken at one minute intervals using a PherStar FS plate reader (BMG labtech) for 5 min prior to and 30 min following the addition of the indicated test compounds. Net BRET responses were calculated as the 535/475 ratio after correcting for both the well baseline and test compound vehicle response. BRET data was then reported as the area under the Net BRET curve for the 30 min following test compound addition.

Primary neurons

Primary cortico-hippocampal neuron cultures from wild type or M1-PD mouse strains were cultured for 7 days in white half area 96-well plates. After 7 days in culture neurons were transfected using lipofectamine MessengerMAX transfection reagent (ThermoFisher) according to the manufacturer instructions with mRNA coding for Nluc-β-arrestin-2 and mNG-CAAX in a 1:5 ratio. For positive control experiments, neurons were also transfected with mRNA coding for the human free fatty acid 4 receptor, which we have previously shown to interact strongly with β-arrestin-2 (3). After transfection, Neurons were cultures for an additional 24h before using in arrestin recruitment assays. Neurons were washed twice with HBSS-H then incubated HBSS-H for 30 min at 37°C. NanoGlow substrate (Promega) was added to a final 1:800 dilution before plates were incubated a further 10 minutes. BRET measurements were then taken using a PheraStar FS plate reader (BMG Labtech), recording the donor emission at 475 nm and acceptor

excitation at 535 nm at one minute intervals. After five minutes, either vehicle or test compounds were added (100μM Ach, or 10μM TUG-891 for positive controls) before continuing to take measurements for an additional 30 min. BRET ratios were calculated as the emission at 535/475, corrected for basal BRET prior to compound addition and expressed as the net BRET above vehicle treatment. The area under the curve (AUC) was then calculated over the full 30 min of compound addition.

BRET early endosome association assay

Internalization of M1-WT and M1-PD was assessed using a bystander BRET assay designed to measure the translocation of M1 receptor to early endosomes (2). HEK293T cells were co-transfected with: 1) a plasmid encoding wild-type or PD mouse M1 fused at its C terminal to Nluc; and 2) a plasmid encoding mNG fused at its C terminal to the FYVE domain of endofin. A DNA weight transfection ratio of 1:2 was used for Nluc-tagged M1 receptor to mNG-FYVE. 24 h after transfection cells were transferred to white 96-well plates and then plates were cultured a further 24 h prior to the assay. For the assay, plates were washed twice with HBSS-H, before incubating in HBSS-H for 30 min at 37 °C. Coelenterazine 400a was added to a final concentration of 5 μM, plates were then incubated a further 10 min. BRET measurements were taken using a PherStar FS plate reader, measuring luminescent emission at 535 and 475 nm at 2 min intervals for 6 min prior to addition of test compounds, and a further 1 h after addition of test compounds. Net BRET responses were calculated as the 535/475 ratio after

correcting for both the well baseline and test compound vehicle response. BRET data was then reported as the area under the Net BRET curve for the 60 min following test compound addition.

BRET Gq activation assay

Activation of Gq by mM1-Wt and mM1-PD was assessed using the 'TruPath' BRET G protein biosensors (4). Cells were transfected with a 0.2:1:1:1 ratio of plasmids encoding: 1. mM1-Wt or mM1-PD, 2. Gq with an internal Rluc2 tag as described in (4) 3. Gγ9, and 4. Gβ3. 24 hours following transfection, HEK293T cells were plated in white half-area 96-well plates coated with 20 μL/well of 5 μg/mL poly-D-lysine. The plate was then cultured a further 24h prior to the assay. Cells were washed with Hank's Balanced Salt solution supplemented with 20 mM HEPES (HBSS-H). For endpoint studies, coelenterazine 400 A substrate (NanoLight Technologies) was added to a final concentration of 5 μM before ACh was added at the indicated concentrations. 5 min after the addition of Ach the bioluminescent emissions at 385-90nm and 525-90nm were measured using a CLARIOstar microplate reader (BMG Labtech). The BRET ratio of 525/385 emission was taken and responses were expressed as the fold change in BRET ratio over vehicle treatment. For Gq activation kinetic studies, the Rluc substrate Prolume Purple (NanoLight Technologies) was added to a final concentration of 5 μM. Bioluminescent emission at 525 and 385 was then measured in a CLARIOstar microplate reader at 1s intervals for 10s prior to the addition of ACh then a further

50s. Net BRET responses were calculated as the 525/385 ratio after correcting for both the well baseline and test compound vehicle response.

Bias Factor calculations

Bias factors were calculated by first fitting an operation model (5) to concentration response data in the arrestin or Gq activation assays to obtain τ/K_A values for ACh at both M1-WT or M1-PD. $\Delta\tau/KA_{PD}$ values were obtained by subtracting the τ/K_A obtained for the M1-WT from the value obtain for M1-PD from the same assay. $\Delta\Delta\tau/K_A$ values were then calculated as the difference between the $\Delta\tau/KA_{PD}$ the two assays. The antilog(10) of this value was then reported as the 'bias factor'.

RNA extraction

RNA extraction was performed immediately following tissue lysis using the Qiagen lipid tissue RNeasy Plus Mini kit as per manufacturer instructions. Briefly, following homogenisation in RLT Buffer containing 10% β-mercaptoethanol, the homogenate was centrifuged at 10,000 x g for 30 sec in a gDNA eliminator column. The aqueous phase containing RNA was collected and mixed with 70% ethanol, then applied to an RNeasy Mini spin column and subjected to centrifugation at 8000 x g for 15 sec at room temperature. The flow-through was discarded and the column was washed with guanidine containing stringent wash buffer, then washed with mild wash buffer, centrifuging for 2 min after the final wash to remove residual ethanol. RNA was eluted in 40 µl nuclease-free water.

Determination of RNA concentration

RNA concentration was determined by measuring absorbance at 260 nm using a NanoDrop spectrophotometer. RNA purity was assessed using the A230/A260 and A260/A280 ratios, with ratios of 2–2.2 considered pure. RNA was stored at –80°C until use.

qRT-PCR

For cDNA synthesis, 1 µg total RNA template per reaction was used using High Capacity cDNA Reverse Transcription Kit (Agilent). RNAase-free water (total 4.2 µl), 2 µl 10x RT buffer, 1 µl RT enzyme, 0.8 µl 25x dNTP Mix (100 mM) and 2µl 10x RT random primers were mixed together and incubated for 10 min at 25°C, followed by 120 min at 37°C, followed by 5 min at 85°C. Samples were then chilled at 4°C. Each reaction included control reactions in the absence of RT enzyme (-RT control). cDNA samples were stored at -20°C until qRT-PCR was performed. Each reaction was conducted in a total volume of 14 µl containing: 7 µl SYBR Green Master Mix, 1.4 µl primers (10 µM stock), 4.2 µl water and 1 µl cDNA (or –RT sample). Each reaction was performed in triplicate.

QuantiTect Primer Assays (Qiagen) were used for all the genes analysed. Sequences of the QuantiTect Primer Assays are not provided but the approximate location of primers within a specific gene can be viewed on the Product Detail of the manufacturer's website.

Primer Template	Primer assay name	Source and P/N
CD86 (mouse)	Mm_Cd86_1_SG	(Qiagen, P/N: QT01055250)
Chrm1 (mouse)	Mm_Chrm1_1_SG	(Qiagen, P/N: QT00282527)
Gfap (mouse)	Mm_Gfap_1_SG	(Qiagen, P/N: QT00101143)
IL-1β (mouse)	Mm_Il1b_2_SG	(Qiagen, P/N: QT01048355)
IL-4 (mouse)	Mm_Il4_1_SG	(Qiagen, P/N: QT00160678)
IL-6 (mouse)	Mm_Il6_1_SG	(Qiagen, P/N: QT00098875)
IL-10 (mouse)	Mm_Il10_1_SG	(Qiagen, P/N: QT00106169)
IL-11 (mouse)	Mm_Il11_1_SG	(Qiagen, P/N: QT00122122)
IL-13 (mouse)	Mm_Il13_1_SG	(Qiagen, P/N: QT00099554)
Prion protein (mouse)	Mm_Prnp_1_SG	(Qiagen, P/N: QT00101080)
α-tubulin (mouse)	Mm_Tuba1b_1_SG	(Qiagen, P/N: QT00198877)

Sample preparation for Western blotting

Animal tissues were snap frozen in liquid nitrogen at sacrifice and stored at −80°C. For preparation of tissue homogenates, frozen tissue was homogenised in ice-cold RIPA buffer (50 mM Tris-HCl pH 8, 150 mM NaCl, 0.5% (w/v) Na-deoxycholate, 1% (v/v) IGEPAL CA-630, 0.1% (v/v) SDS) including protease and phosphatase inhibitor cocktails (cOmplete™, Mini Protease Inhibitor Cocktail # 11836153001, cOmplete™, Mini Protease Inhibitor Cocktail # 11836153001, Sigma Aldrich -- Merck Life Science) using a sonicator at amplitude 3-5 Hz. The homogenates were incubated for 30 minutes at 4°C with end over end rotation and centrifuged at

21,000 x g for 15 minutes. Supernatants were transferred to fresh microcentrifuge tubes and stored at -80°C until use.

For the preparation of membrane extracts, frozen hippocampi and cortices were homogenised by sonication at 3-5 Hz amplitude in 500 µl of T/E buffer (10 mM Tris, 1 mM EDTA, pH 8.0) containing phosphatase and protease inhibitors. Samples were then centrifuged at 10,000 x g for 10 min at 4 °C. The supernatants were mixed with additional 500 µl T/E buffer, and then centrifuged at 15,000 x g for 1 hour at 4 °C. The pellets were then solubilised in 400 µL of RIPA buffer including phosphatase and protease inhibitors and incubated for at least 2 hours at 4°C with end over end rotation. After centrifugation of samples at 14,000 x g for 10 min at 4°C, the supernatants (membrane extracts) were transferred to fresh microcentrifuge tubes and stored at -80°C until use.

Protein concentrations were determined by using the Micro BCA protein assay reagent kit according to the manufacturer's instructions.

Western blotting

Following preparation as described above, samples were incubated with Laemmli loading buffer containing 5% β-mercaptoethanol. Membrane extracts were incubated for 30 min at 37°C, whereas brain homogenates were heated at 95°C for 5 min, and briefly spun down before loading the gel. Samples (10 µg) were run in either 7.5% (>60 kDa) or 12% (<60 kDa), -SDS-PAGE minigels at 60-150 V in

Tris-glycine SDS running buffer (Serven biotech ltc, cat 20-6400-50). Nitrocellulose membranes were equilibrated in transfer buffer, before assembly the blot sandwich. The transfer was performed for 2 hours at constant 60V. Then, membranes were blocked for 30-45 min in 5% (w/v) fat-free milk (or bovine serum albumin when probing phosphorylated targets) in TBS-T (0.1% tween-20 in TBS at pH 7.4) at room temperature. Membranes were then incubated with the respective primary antibody at 4°C overnight or room temperature for 2 hours. The following primary antibodies were used: mouse anti-alpha Tubulin [DM1A] (Abcam, #ab7291, 1:10,000), rabbit anti-Apolipoprotein E [EPR19378] (Abcam, #ab183596, 1:1000), goat anti-Clusterin (R&D Systems, #AF2747, 1:1000), rabbit anti-Galectin 1 [EPR3205] (Abcam, #ab108389, 1:1000), mouse anti-GFAP (Sigma-Aldrich, #G3893, 1:5000), rat HA-Biotin [3F10] (Roche, #12158167001, 1:500), rabbit anti-Sodium Potassium ATPase [EP1845Y] (Abcam, #ab76020, 1:10,000); mouse anti-PrP [8H4] (Abcam, #ab61409, 1:1000), goat anti-SerpinA3N (R&D Systems, #AF4709, 1:1000), mouse anti-Vimentin (R&D Systems, #MAB21052, 1:1000), mouse anti-Phospho-p44/42 MAPK (Erk1 (Y204)/Erk2 (Y187); Cell Signaling, #5726, 1:1000), rabbit anti-p44/42 MAPK (Erk1/2; Cell Signaling, #9102, 1:1000). Then, membranes were incubated with the respective secondary antibodies (1:10,000) for one hour at room temperature: donkey anti-mouse (LI-COR Biotechnology, #926-68022 or #926-32212), donkey anti-Rabbit (LI-COR Biotechnology, #926-32213), donkey anti-Rat (LI-COR Biotechnologies, #926-32219). Proteins were visualised using LICOR Odyssey SA scanner using the appropriate lasers.

Immunohistochemistry

Following heat-induced epitope retrieval, sections were washed in TBS + 0.1% triton x-100, and blocked overnight at 4°C in TBS, 0.1% triton X-100, 10% goat serum and 1% BSA. Incubation with primary antibodies (anti-GFAP, Sigma-Aldrich, P/N: G3893; anti-Iba1, Thermo Fisher, P/N: PA5-27436) was conducted in blocking buffer overnight at 4°C or for two hours at room temperature. Following three washes, slides were incubated with Alexa Fluor fluorescent secondary antibodies for 2 hours at room temperature in blocking buffer. Following three washes, slices were mounted on glass slides using VECTASHIELD HardSet Antifade Mounting Medium with DAPI, let dry overnight at 4°C and sealed using nail varnish. All images were taken using LSM 880 confocal laser scanning microscope (Zeiss).

Data analysis

Data statistical analyses were carried out using GraphPad Prism 9 software. Functional concentration–response curves were fitted according to a four-parameter logistic equation to determine basal and maximal responses, log half maximal effective concentration (EC_{50}) and slope.

Quantification of band intensity was achieved by measuring the median pixel intensity (arbitrary units) using Image Studio Lite (Version 5.2), a LICOR-recommended software for blot analysis. The background signal was automatically

corrected by this software. Band intensity corresponding to the protein of interest was always normalised to housekeeper protein, typically α-tubulin. Densitometry measurements were assumed to be normally distributed and were statistically compared using two-way ANOVA with Sidak multiple comparisons.

For qRT-PCR data analysis, C_T values were compared with a suitable internal control (housekeeper gene), typically α-tubulin for normalisation and to calculate ΔC_T values (ΔC_T = C_T of housekeeper − C_T of test gene). ΔC_T values were then compared with the control conditions, typically for the M1-WT controls, to calculate the $\Delta\Delta C_T$. All the data is finally expressed as $2^{-\Delta\Delta CT}$ to get the expression fold change, and compared using two-way ANOVA with Sidak multiple comparisons.

Burrowing data were compared using mixed-effects model with uncorrected Fisher's LSD test. Symptom onset and survival curves were analysed with a Gehan-Breslow-Wilcoxon test.

SI References

1. R. Schrage *et al.*, The experimental power of FR900359 to study Gq-regulated biological processes. *Nature Communications* **6**, 10156 (2015).

2. Y. Namkung *et al.*, Monitoring G protein-coupled receptor and beta-arrestin trafficking in live cells using enhanced bystander BRET. *Nat Commun* **7**, 12178 (2016).

3. B. D. Hudson *et al.*, The pharmacology of TUG-891, a potent and selective agonist of the free fatty acid receptor 4 (FFA4/GPR120), demonstrates both potential opportunity and possible challenges to therapeutic agonism. *Mol Pharmacol* **84**, 710-725 (2013).

4. R. H. J. Olsen *et al.*, TRUPATH, an open-source biosensor platform for interrogating the GPCR transducerome. *Nat Chem Biol* **16**, 841-849 (2020).

5. E. T. van der Westhuizen, Breton, B., Christopoulos, A., and Bouvier, M, Quantification of ligand bias for clinically relevant beta2-adrenergic receptor ligands: implications for drug taxonomy. *Mol Pharmacol* (2014).

Supplementary Figure 1 Arrestin recruitment and receptor internalisation is impaired when phosphorylation sites are removed from the M1-receptor.

(A-B) Time-course of ACh-stimulated translocation of β-arrestin-2 to the cell membrane in HEK293T cells transfected with M1-WT **(A)** or M1-PD **(B)** assessed by bystander BRET. Net BRET response after 5 minutes stimulation with ACh is shown in **C**. Data are expressed as mean ± SEM of 5 independent experimented performed in triplicates. **(D-E)** Time course of translocation of M1-WT **(D)** and M1-PD **(E)** to early endosomes in response to ACh treatment, assessed through a bystander BRET assay. Net BRET response after 30 minutes stimulation with ACh is shown in **F**. Data are expressed as mean ± SEM of 5 independent experimented performed in triplicates. **(G-H)** Time course of ACh-stimulated Gαq activation by the M1-WT **(G)** or M1-PD **(H)** measured by a decrease in BRET. Net BRET response at 10 seconds following ACh-stimulation is shown in **I**. Data are expressed as means ± S.E.M. of 3 independent experiments performed in triplicate.

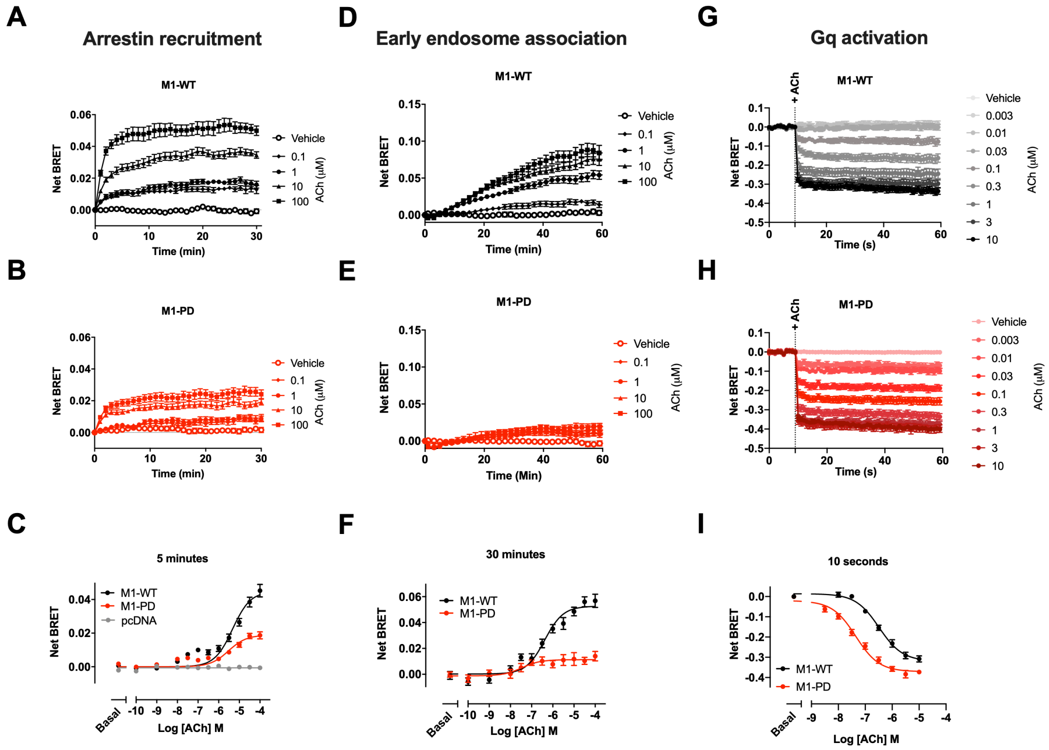
A
Arrestin recruitment
M1-WT
Net BRET
Time (min)
Vehicle
0.1
1
10
100
ACh (μM)
B
M1-PD
C
5 minutes
M1-WT
M1-PD
pcDNA
Basal
Log [ACh] M
D
Early endosome association
E
Time (Min)
F
30 minutes
G
Gq activation
+ ACh
Time (s)
0.003
0.01
0.03
0.3
3
H
I
10 seconds

Supplementary Figure 2 ERK1/2 signalling mediated by the M1-receptor is mediated by Gq protein-dependent signalling. Time course of pERK signalling was performed in HEK293T cells transfected with M1-WT or M1-PD stimulated with ACh in presence or absence of the Gq protein inhibitor FR900359 (56). Data are expressed as means ± S.E.M. of 3 independent experiments performed in duplicate (n=3).

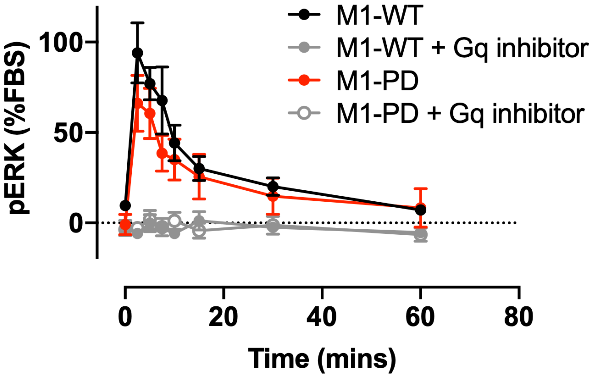

Supplementary Figure 3 M1 receptors are equivalently expressed in M1-WT and M1-PD mice. (A) Quantitative analysis of M1-receptor RNA expression in M1-WT, M1-PD and M1-KO cortex and hippocampus. Data is expressed as means ± S.E.M of a ratio of α-tubulin RNA expression relative to M1-WT (n=4 mice). **(B)** Western blot analysis of solubilsed membranes from M1-WT, M1-PD and M1-KO cortex and hippocampus for detection of HA-tagged receptors. Data shown are from three separate mice for the M1-WT and M1-PD genotypes. Na^+/K^+ ATPase expression was used as a loading control. **(C)** Bands from (**B**) were analysed for (semi)quantification of receptor expression and data is expressed as a ratio of Na^+/K^+ ATPase expression relative to the M1-WT (n=3 mice).

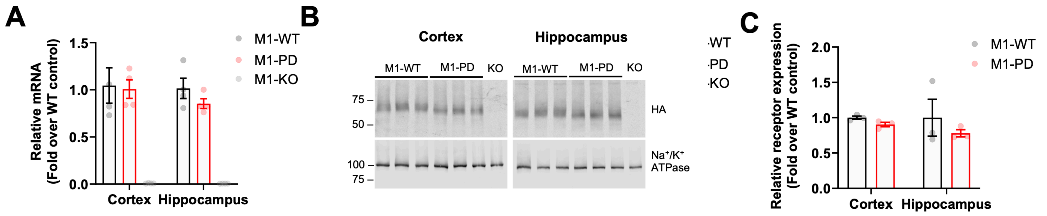

Supplementary Figure 4 Arrestin recruitment to transiently transfected FFA4 receptors in M1-WT and M1-PD neurons is equivalent. (A) Time-course of TUG-891 (10 μM)-stimulated translocation of β-arrestin-2 to the cell membrane in primary hippocampal-cortical neurons prepared from M1-WT or M1-PD mice and transfected with human FFA4 receptor. **(B)** BRET (AUC) response after 30 minutes stimulation with TUG-891. Data are expressed as mean ± SEM of 4-6 independent experiments performed in triplicates.

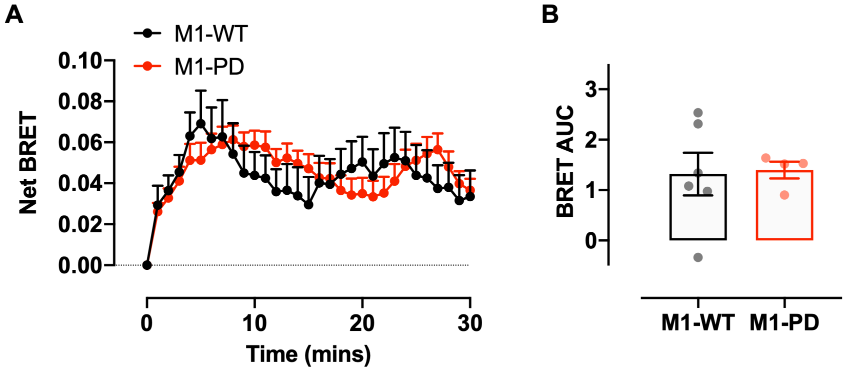

Supplementary Figure 5 Control- and prion infected M1-PD mice show equivalent receptor expression and signalling compared to M1-WT mice. (A) Quantitative analysis of M1-receptor RNA expression in the cortex and hippocampus of 16 weeks post-inoculation (w.p.i.) control- and prion-infected, M1-WT and M1-PD mice. Data is shown as means ± S.E.M of a ratio of α-tubulin RNA expression relative to M1-WT (n=4 mice). **(B)** Solubilised membranes from 16 w.p.i. control- and prion-infected M1-WT and M1-PD cortex and hippocampus were probed in a Western blot for the expression of M1-receptor using an antibody against the HA tag. Data shown are two separate mice for the control- and prion-infected M1-WT and M1-PD genotypes. M1-KO was also probed as negative control, and Na^+/K^+ ATPase expression was used as a loading control. **(C)** Band analysis from the western blot **(B)** was conducted for (semi)quantification of receptor expression and data is expressed as means ± S.E.M. of ratios of Na^+/K^+ ATPase expression relative to the M1-WT (n=2 mice).

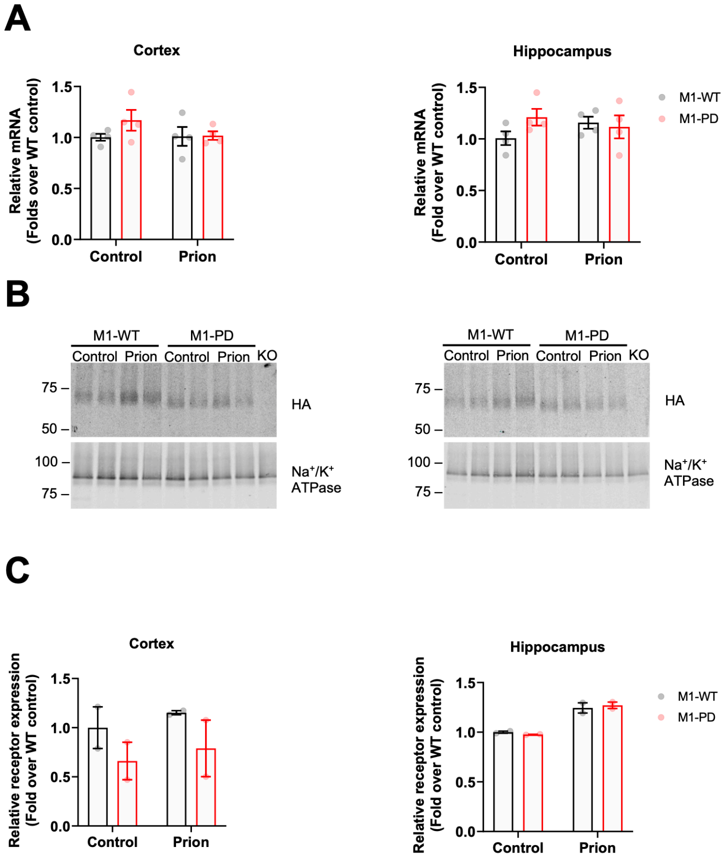
A
Cortex
Relative mRNA (Folds over WT control)
Control
Prion
Hippocampus
Relative mRNA (Fold over WT control)
M1-WT
M1-PD
B
M1-WT
M1-PD
Control
Prion
KO
75
50
100
HA
Na⁺/K⁺ ATPase
C
Cortex
Relative receptor expression (Fold over WT control)
Hippocampus

Supplementary Figure 6 Prion infected M1-PD mice show accelerated appearance of disease markers in the cortex compared to M1-WT mice. Lysates were prepared from the cortex of control- or prion infected M1-WT and M1-PD mice at 16- and 18 w.p.i. and Western blot analysis was used to analyse the expression of a panel of pathological markers. **(A)** Lysates were incubated in the presence or absence of proteinase K prior to Western blot to detect non-digested scrapie prion protein (PrP_{sc}) and total prion protein (PrP_{tot}), respectively. Band analysis for PrP_{sc} and PrP_{tot} expression in **(B)** is shown as means ± S.E.M. of a ratio of α-tubulin expression (n=3). **(C)** Apolipoprotein-E (APO-E), serpinA3N, clusterin and galectin-1 were detected in the cortex and band analysis is shown in **(D)** as means ± S.E.M. of a ratio of α-tubulin expression relative to control-infected M1-WT (n=3 mice). Data were analysed using a two-way ANOVA with Sidak multiple comparisons where *P<0.05, **P<0.01(M1-WT vs. M1-PD).

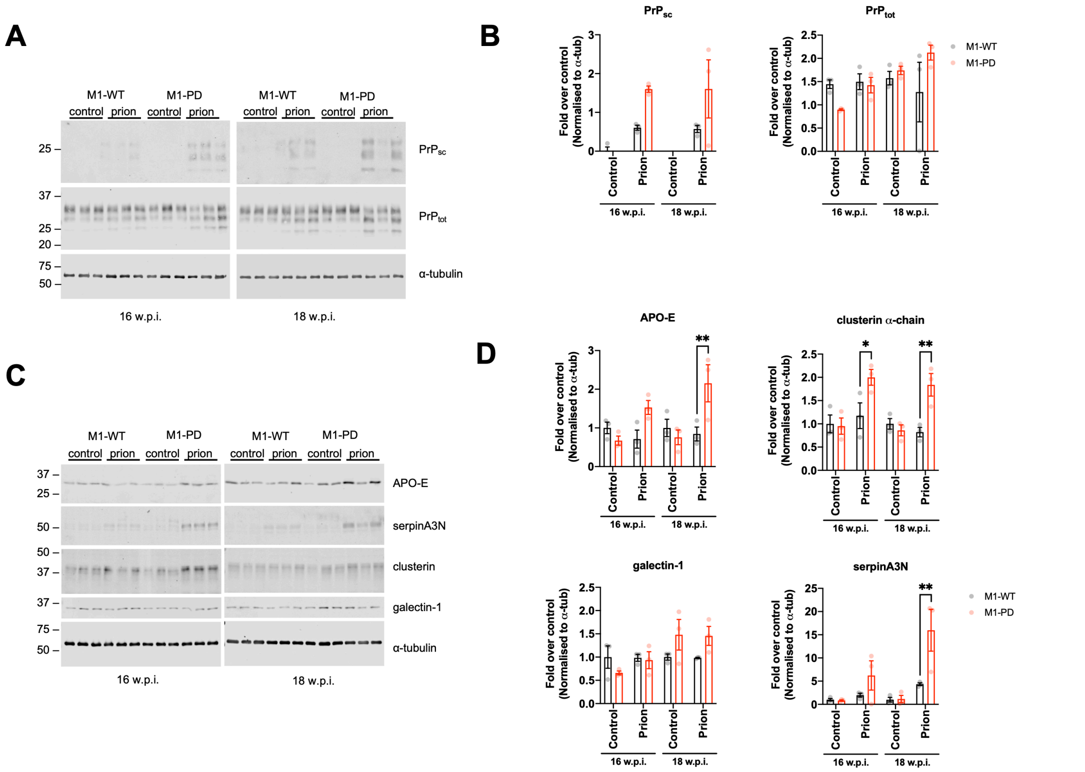

A
B
C
D
M1-WT
M1-PD
control
prion
PrPsc
PrPtot
α-tubulin
16 w.p.i.
18 w.p.i.
APO-E
serpinA3N
clusterin
galectin-1
clusterin α-chain
Fold over control (Normalised to α-tub)
Control
Prion
**
*

Supplementary Figure 7 Prion protein (PrP) mRNA expression is unchanged in the hippocampus and cortex of M1-PD mice compared to M1-WT. Quantitative RT-PCR showing the expression of PrP RNA in the cortex and hippocampus of M1-WT and M1-PD mice. Data are expressed as a ratio of α-tubulin RNA expression (n=4 mice).

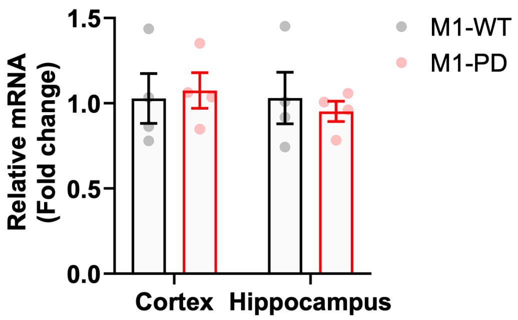

Supplementary Figure 8 Control or prion infected M1-PD mice show similar expression and activation of Extracellular signal-regulated kinase (ERK) to M1-WT mice. **(A)** Western blots to determine the presence of ERK1/2 and phosphorylated ERK1/2 (Y204)/Y187) in the cortex and hippocampus during prion disease was conducted on lysates prepared from control or prion-infected, M1-WT or M1-PD mice at 18 w.p.i.. α-tubulin (α-tub) antibody was used for loading control. Band analysis for ERK1/2 and P-ERK1/2 in **(B)** is expressed as means ± S.E.M. of a ratio of α-tubulin expression relative to control-infected M1-WT (n=3 mice). Statistical analysis performed is two-way ANOVA with Sidak multiple comparisons.

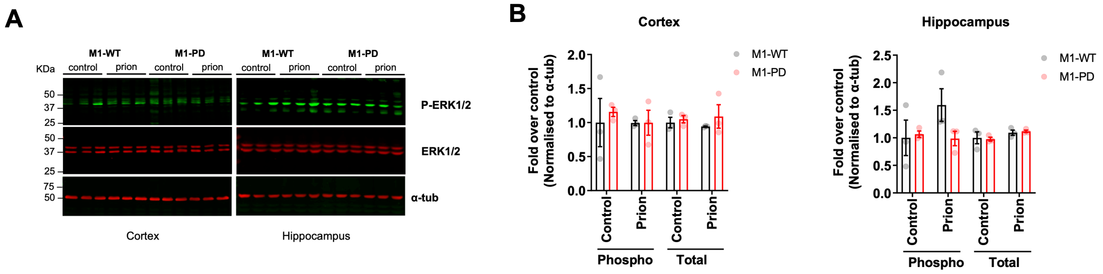

Supplementary Figure 9 Neuroinflammation is exacerbated in the cortex of prion infected M1-PD mice compared to M1-WT controls. (A) mRNA levels of GFAP and CD86, markers of astrocytes and microglia respectively, were quantified using quantitative RT-PCR of cortex from control- or prion-diseased M1-WT or M1-PD mice at 16 weeks post inoculation (w.p.i.). Data is expressed as means ± S.E.M. of a ratio of α-tubulin RNA expression relative to M1-WT (n=4 mice). **(B-C)** Astrogliosis in the cortex was assessed using Western blot analysis of lysates prepared from control- or prion-infected mice at 16- and 18 w.p.i. Lysates were probed for astrocytic markers GFAP and vimentin (vim), and α-tubulin (α-tub) antibody was used as a loading control. **(C)** Band analysis for each blot was performed and data is shown as means ± S.E.M. of a ratio of α-tubulin relative to control M1-WT (n=3 mice). **P*<0.05, two-way ANOVA Sidak multiple comparisons (M1-WT vs. M1-PD). **(D)** Immunohistochemical staining for GFAP and Iba-1 in the cortex of control- and prion infected M1-WT and M1-PD mice at 16 w.p.i. The nuclei were stained blue with DAPI. Scale bar 100 µm. **(E)** Quantitative RT-PCR showing the expression of pro-inflammatory (TNF-α, IL-1β, IL-6) cytokines in the cortex of control- and prion infected M1-WT and M1-PD mice at 16 w.p.i. Data are expressed as a ratio of α-tubulin RNA expression relative to control M1-WT (n=4 mice). Data were analysed using two-way ANOVA with Sidak multiple comparisons, where **P*<0.05, ***P*<0.01 (M1-WT vs. M1-PD).

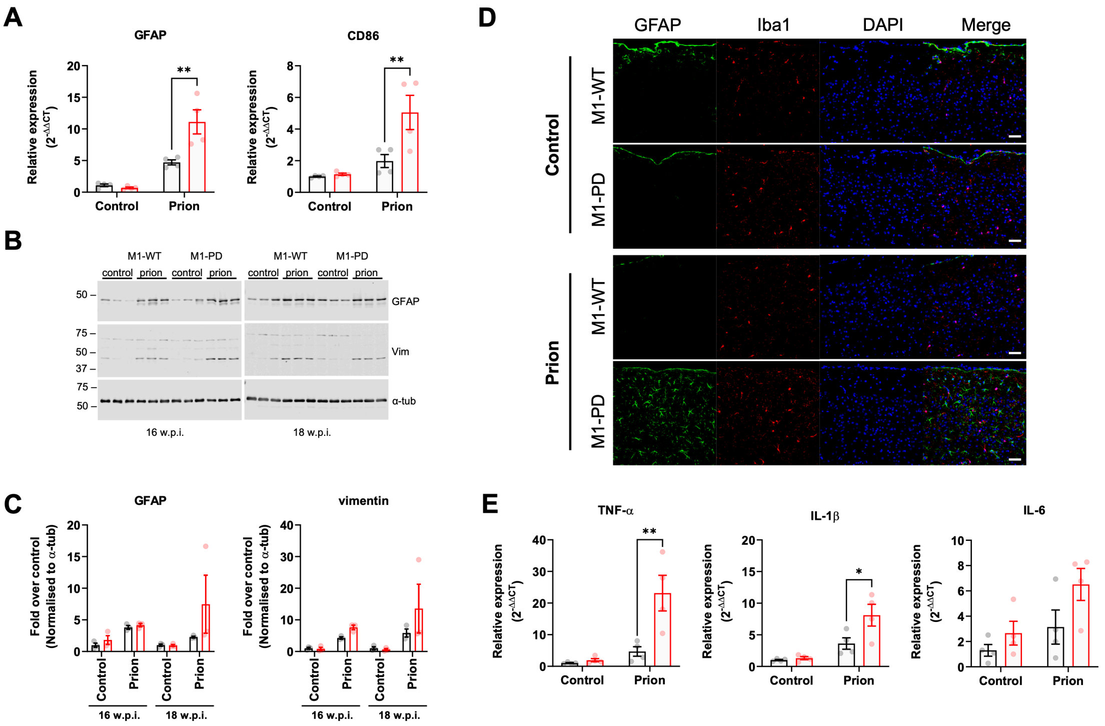
A
GFAP
CD86
Relative expression (2^-ΔΔCT)
Control
Prion
**
B
M1-WT
M1-PD
control
prion
GFAP
Vim
α-tub
16 w.p.i.
18 w.p.i.
C
GFAP
vimentin
Fold over control (Normalised to α-tub)
16 w.p.i.
18 w.p.i.
D
GFAP
Iba1
DAPI
Merge
Control
Prion
M1-WT
M1-PD
E
TNF-α
IL-1β
IL-6
*

Supplementary Figure 10 Expression of anti-inflammatory cytokines in control- and prion infected M1-WT and M1-PD mice. Quantitative RT-PCR showing the expression of anti-inflammatory cytokines, **(A-B)** IL-4, **(C-D)** IL10, **(E-F)** IL-11 and **(G-H)** IL-13 in the cortex **(A, C, E, G)** and hippocampus **(B, D, F, H)** of control- or prion diseased M1-WT or M1-PD mice at 16 w.p.i. Data are expressed as a ratio of α-tubulin RNA expression (n=4 mice).

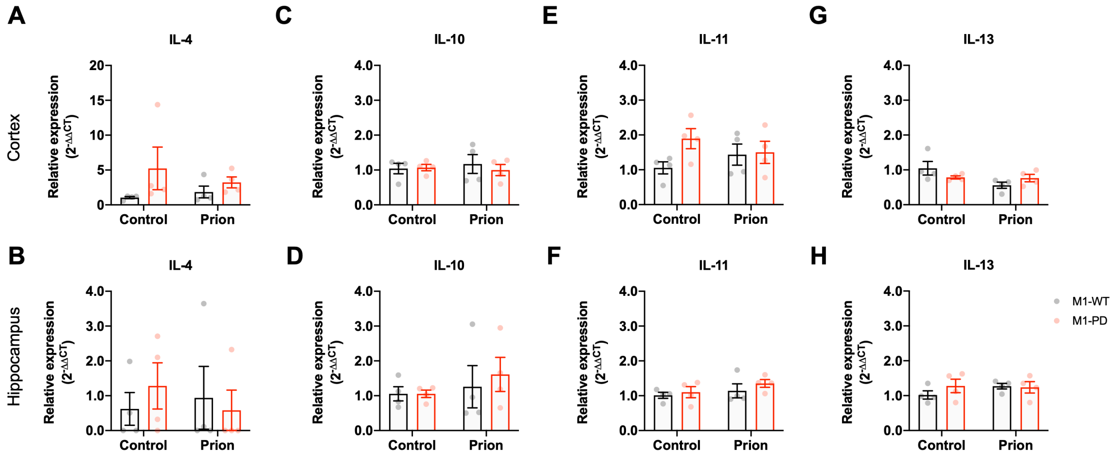

Supplementary Figure 11 Expression of neuroinflammatory markers and cytokines in M1-WT and M1-PD mice. (A-B) Quantitative RT-PCR showing the expression of **(A)** GFAP and **(B)** CD86, markers of astrocytes and microglia respectively, in the cortex or hippocampus of M1-WT or M1-PD mice. **(C-I)** Quantitative RT-PCR showing the expression of pro-inflammatory cytokines **(C)** TNF-α, **(D)** IL-1β and **(E)** IL-6, and anti-inflammatory cytokines **(F)** IL-4, **(G)** IL-10, **(H)** IL-11 and **(I)** IL-13 in the cortex or hippocampus of M1-WT or M1-PD mice. Data are expressed as a ratio of α-tubulin RNA expression (n=4 mice).

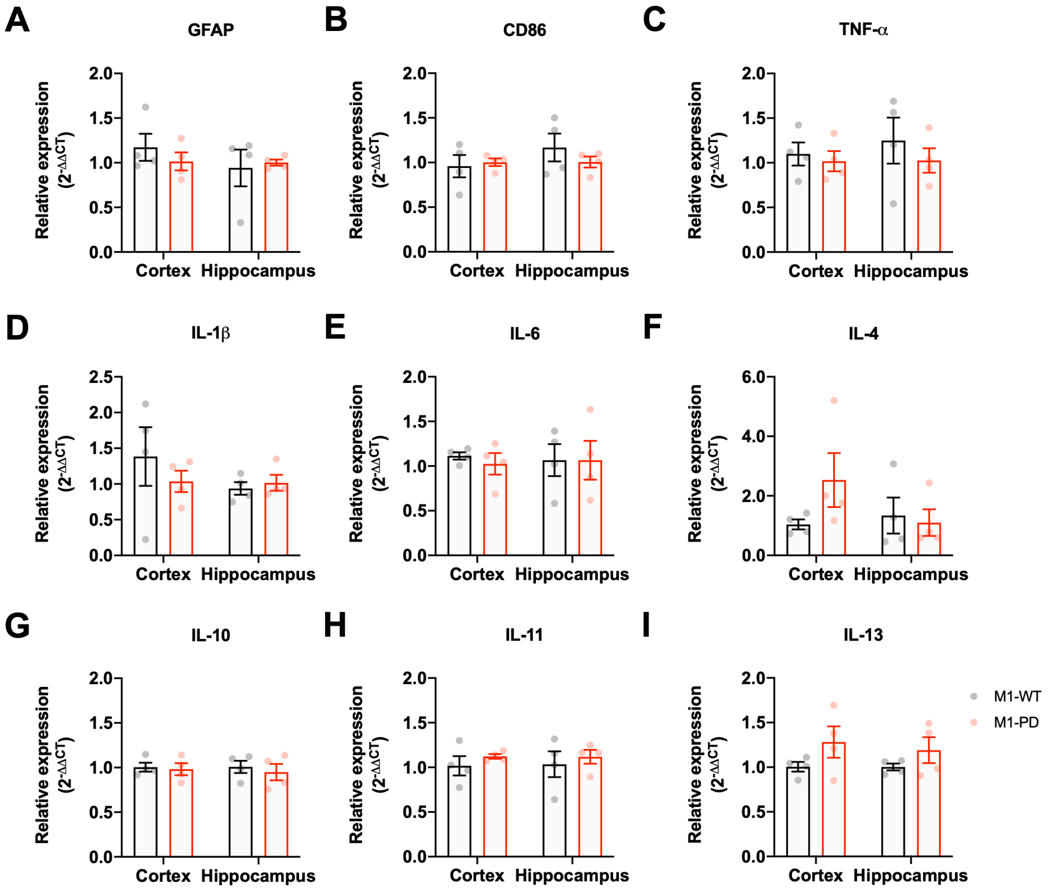

Supplementary Figure 12 Removal of M1-receptor phosphorylation sites accelerates the onset of prion disease indicators. Symptomatic mice were analysed according to the appearance of recognised early disease indicators including **(A)** subdued (n=23), **(B)** intermittent generalised tremor (n=8-15), **(C)** erect penis (n=5-6), **(D)** rigid tail (n=16-19), **(E)** unsustained hunched posture (n=13-14), and **(F)** mild loss of coordination (n=11-12). Curves were analysed with a Gehan-Breslow-Wilcoxon test, where $^{**}P < 0.01$; $^{****}P < 0.0001$.

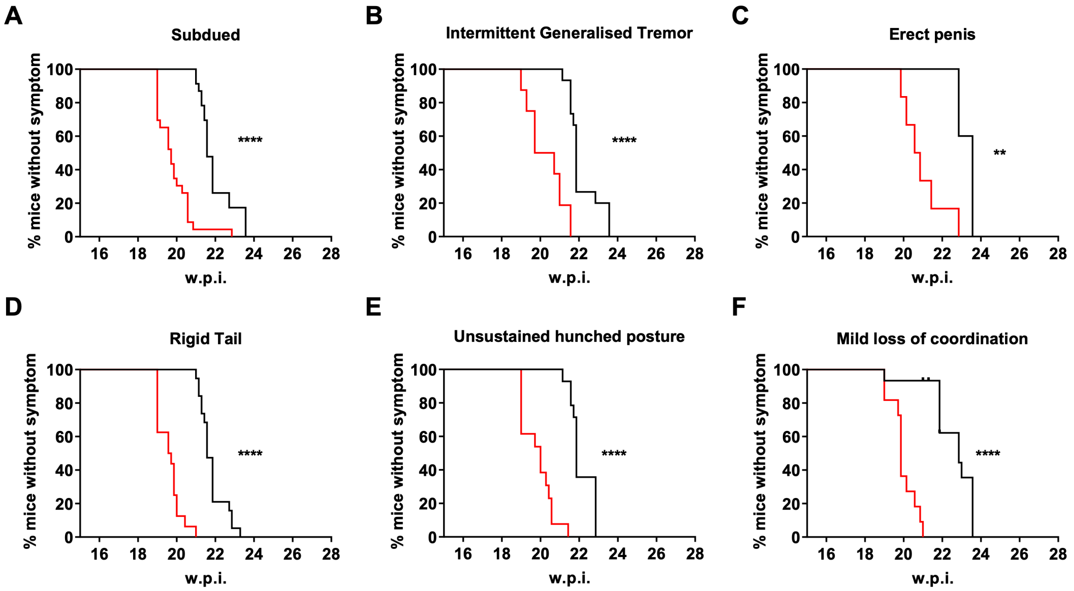

Supplement: Supplementary File [file pnas.2107389118.sapp.pdf]
